# Adversarial Autoencoder and Multi-Armed Bandit for Dynamic Difficulty Adjustment in Immersive Virtual Reality for Rehabilitation: Application to Hand Movement

**DOI:** 10.3390/s22124499

**Published:** 2022-06-14

**Authors:** Kenta Kamikokuryo, Takumi Haga, Gentiane Venture, Vincent Hernandez

**Affiliations:** 1 Department of Mechanical Systems Engineering, Tokyo University of Agriculture and Technology, Tokyo 184-0012, Japan; kenta.kamikokuryo0708@gmail.com (K.K.); takumihaga629@gmail.com (T.H.); 2 Department of Mechanical Engineering, The University of Tokyo, Tokyo 113-8654, Japan; venture@cc.tuat.ac.jp; 3 Surfclean Inc., Sagamihara 252-0131, Kanagawa, Japan

**Keywords:** machine learning, reinforcement learning, multi-armed bandit, immersive virtual reality, dynamic difficulty adjustment, end effector

## Abstract

Motor rehabilitation is used to improve motor control skills to improve the patient’s quality of life. Regular adjustments based on the effect of therapy are necessary, but this can be time-consuming for the clinician. This study proposes to use an efficient tool for high-dimensional data by considering a deep learning approach for dimensionality reduction of hand movement recorded using a wireless remote control embedded with the Oculus Rift S. This latent space is created as a visualization tool also for use in a reinforcement learning (RL) algorithm employed to provide a decision-making framework. The data collected consists of motions drawn with wireless remote control in an immersive VR environment for six different motions called “Cube”, “Cylinder”, “Heart”, “Infinity”, “Sphere”, and “Triangle”. From these collected data, different artificial databases were created to simulate variations of the data. A latent space representation is created using an adversarial autoencoder (AAE), taking into account unsupervised (UAAE) and semi-supervised (SSAAE) training. Then, each test point is represented by a distance metric and used as a reward for two classes of Multi-Armed Bandit (MAB) algorithms, namely Boltzmann and Sibling Kalman filters. The results showed that AAE models can represent high-dimensional data in a two-dimensional latent space and that MAB agents can efficiently and quickly learn the distance evolution in the latent space. The results show that Sibling Kalman filter exploration outperforms Boltzmann exploration with an average cumulative weighted probability error of 7.9 versus 19.9 using the UAAE latent space representation and 8.0 versus 20.0 using SSAAE. In conclusion, this approach provides an effective approach to visualize and track current motor control capabilities regarding a target in order to reflect the patient’s abilities in VR games in the context of DDA.

## 1. Introduction

The main goal of motor rehabilitation is to improve motor control abilities through intensive and repetitive intervention in order to improve the patient’s quality of life. Regular evaluations are necessary to measure the effectiveness of the therapy on the patient and to adapt it if necessary. However, it is a tedious task, and it would be interesting to find a way to facilitate the rehabilitation evaluations with simplified metrics. Furthermore, it is important to provide tools that can help the patient to continue the rehabilitation process at home. Supporting the analysis process with various sensors could be very interesting, as the data can be used for automated and remote monitoring [1,2]. In addition, given the time-consuming and repetitive aspect of conventional therapy, some complementary approaches are needed to keep the patient motivated [3].

Virtual reality (VR) refers to a technology used to interact with a simulated environment that attempts to recreate certain aspects of the real world to engage the user in various actions. VR rehabilitation is a promising and relevant tool as an additional tool to conventional rehabilitation [4,5] that allows the creation of specific environments for therapy that incorporate precise control of the actions performed [3]. Various studies that have compared rehabilitation with and without game integration have shown that it significantly improves outcomes if VR games are involved [6]. The enjoyment of VR games can help promote patient engagement by reducing boredom from task repetition by providing an alternative to clinical environments [7,8]. Interaction with the virtual environment through motor action allows patients to feel part of a virtual world and can contribute to the rehabilitation process [9]. In addition, through recordings and performance evaluation, it is possible to assess current progress and provide a personalized experience by adjusting the difficulty and scenario of the game according to the patient’s motor control ability [10,11,12].

Specifically, immersive VR is based on a head-mounted display (HMD) that allows participants to be immersed in an audiovisual virtual environment. Interaction with the virtual world is made possible by wireless remote devices held in both hands, allowing an interactive experience with the virtual world. This device is integrated with most HMD systems (e.g., Oculus Rift or HTC Vive) and allows for an accurate assessment of the position of the HMD and the remote control [13,14]. HMD-VR systems are becoming more affordable and can be an attractive tool for home rehabilitation [2].

A more advanced functional assessment of the patient’s performance level can be achieved using motion tracking technology such as IMUs, motion capture systems, or Kinect, to name a few [8]. This can enhance the experience by incorporating visuomotor feedback that aims to represent the movement of the patient’s limb in the virtual world by incorporating an embedded body. Other approaches, such as tactile feedback (vibrations, small forces applied to the skin) or force feedback (resistance movement), can also be considered [8].

To improve the effectiveness of using immersive VR in rehabilitation, the level of difficulty must be adapted to the patient’s current motor control abilities. Indeed, patients may become bored if the game is too easy or frustrated if it is too difficult, which may reduce their engagement in the game. Changing the motor task to be performed according to the patient’s abilities is important [3]. This should be assessed and adjusted sequentially as the patient is involved in the therapeutic process. Providing an accurate level of challenge during therapy can be a difficult and time-consuming task for healthcare professionals, as it requires continuous analysis of data from previous and new rehabilitation sessions. Therefore, automatic adjustment of the level of challenge is necessary by adapting features such as the rates of specific tasks to be offered at a given time based on motor control capabilities.

Dynamic Difficulty Adjustment (DDA) is defined as a method of adapting the characteristics of a serious game to adjust the level of difficulty based on the player’s skills to provide a good balance of difficulty. According to the flow theory in human–computer interaction [15], the appropriate level of difficulty of a game should depend on the player’s skills [16,17,18].

The concept of DDA in rehabilitation is to provide a continuous adjustment of the difficulty of the online game and adapt to the player’s motor control capability. Furthermore, if the patient’s ability has changed significantly since the last gaming session, the system must adapt quickly. It must constantly adapt to any change, whether it is an improvement or a decrease in performance, in order to maintain the player’s motivation and focus [19]. To customize the difficulty, the DDA can adjust certain features of the game, such as the behavior of the enemies or the game scenario.

Few DDA methods have examined real-time difficulty adjustment using sequential data in the context of VR rehabilitation [11,20,21,22,23]. Our motivation is to propose probabilistic decision-making for adjusting the difficulty based on the patient’s movement in order to dynamically modify the game requirement throughout the sessions. This can reduce the need for direct supervision by a therapist and allow for adaptive and individualized home video game therapy. Most DDA approaches are based on adjusting the game according to the ratio of gains to losses, the time required to complete a specific task, and the remaining life points, to name a few. Such an approach can be improved by taking into account the current level of motor control based on recorded movement, thus integrating an essential part of physical therapy into the decision-making process.

The objective of this paper is twofold. The first is to provide an efficient visualization tool for high-dimensional data by considering an effective dimensionality reduction of the hand motion recorded using a wireless remote control embedded in the Oculus Rift S. The second is to use the reinforcement learning (RL) approach as a basis for DDA based on the multi-armed bandit (MAB) algorithm based on the latent space representation.

A data dimensionality reduction model, called adversarial autoencoder (AAE), is considered to represent each matrix as a single two-dimensional point in a latent space. The original shape of the signal is represented in a lower-dimensional space that preserves the similarity between the motions. This allows for the creation of a simplified visualization that allows the current motion to be represented among other motions and helps detect outliers and patterns [24]. In this study, each motion is represented by a single point in a two-dimensional latent space generated by AAE, which is a powerful and efficient approach to perform the dimensionality reduction of nonlinear data [24].

**Figure 1:**
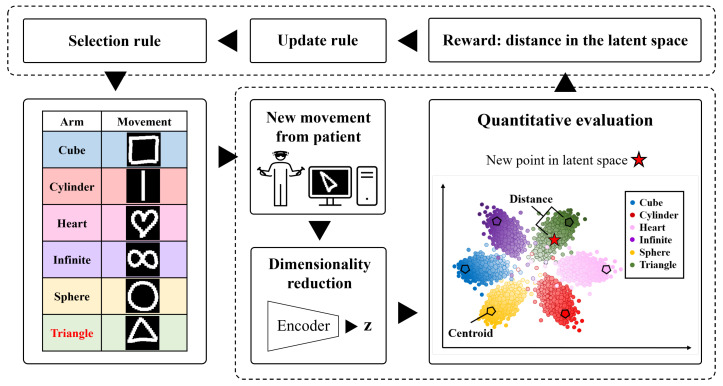
Project Overview. The adversarial autoencoder (AAE) is trained by considering a regularization component to create a set of six Gaussian distributions in a latent space. The trained AAE is then fed with a new sample (with Triangle as an example) and represented in the latent space (red cross). Finally, a distance metric is computed between a target (green centroid) and used as a reward for the MAB algorithm.

Finally, this simplified data representation can be used as a visualization tool to present the current progress of the patient in the latent space with respect to a target (Figure 1). In addition, an RL approach based on the MAB algorithm is used to learn the patient’s current motor abilities by considering the distance in latent space as a simplified metric and applying DDA to the game. To the best of our knowledge, such an approach has not yet been considered.

## 2. Related Work

Most serious VR games use a static difficulty approach requiring manual selection of the difficulty level or a “staircase” progression in which, after the player has completed specific tasks at a given level, the algorithm increases the difficulty of the next level [25,26,27,28]. Predefined difficulty levels have several disadvantages, as the player may choose an inappropriate difficulty level, and the consideration of possible player improvement is not automated. There has been limited research proposing the adjustment of the difficulty of video games in the context of rehabilitation.

Wilms [20] propose an actor-critic method used to create a learning agent that adjusts the difficulty level during cognitive rehabilitation training programs based on response time. They provide an efficient agent that adapts quickly to change, but the threshold parameters of the fitness function between what is considered an easy or difficult task are manually set and task-specific.

Sekhavat [11] propose a multi-periodic reinforcement learning approach for DDA to adjust parameters such as distance, speed, and size based on a periodic assessment at three different intervals during the rehabilitation game. The goal of the game is to hit a specific ball placed on an arc of balls. The participants’ movement is recorded with a Kinect to control the arm of a virtual character. The learning agent adjusts its probability chromosome to increase or decrease the speed, size, and distance of the balls based on success or failure in hitting the ball to adapt the game.

Pirovano et al. [21] have developed a VR game for rehabilitation using the Kinect sensor. The game uses fuzzy logic to monitor joint angles and provide feedback to the patient. If the movement is not performed correctly, the game triggers an alarm during the game. The game adapts the difficulty level using the adaptive Bayesian Quest method based on the win/loss ratio.

Andrade et al. [22] and Andrade et al. [23] proposed an approach for robotic rehabilitation based on an evolutionary algorithm to adjust the difficulty of the game. The game consists of moving a squirrel to catch hazelnuts that fall on a screen. The game is adapted according to the success or failure to reach the hazelnut before it touches the ground and is validated on various simulated movement behaviors.

To the authors’ knowledge, no study has yet used time-series data of hand movements as the basis for DDA in the context of immersive VR rehabilitation to provide a personalized experience. In addition, time-series data is highly dimensional, and reducing the dimensionality of this data is important to produce an RL agent that can learn in a rapidly changing environment.

Hernandez et al. [24] proposed a data dimensionality reduction approach to visualize high-dimensional data from the Wii Balance Board during upper and lower body exercises. This study uses a data dimensionality reduction approach with deep learning models called adversarial autoencoder to visualize time series in a 2D latent space. The study is limited to the visualization approach, and no trajectory identification or learning agents have been proposed.

In this context, it would be interesting to combine two different approaches to address the constraints discussed above. The first relies on reducing the dimensionality of hand movement time series in an immersive VR context to provide a simplified representation of the data. The second uses RL agents based on multi-armed bandits to provide a basis for the decision-making model, with an arm representing information about one movement. A high distance in latent space increases the probability, and a low distance decreases it. In other words, the information about each arm is equivalent to the information about the difficulty for the patient to perform a movement. Such an RL model must be able to adapt to a rapidly changing environment and take into account the uncertainty associated with untested action over time. These aspects are important in the field of rehabilitation, as a patient’s condition may change abruptly.

## 3. Materials and Methods

### 3.1. Experiments

**Figure 2:**
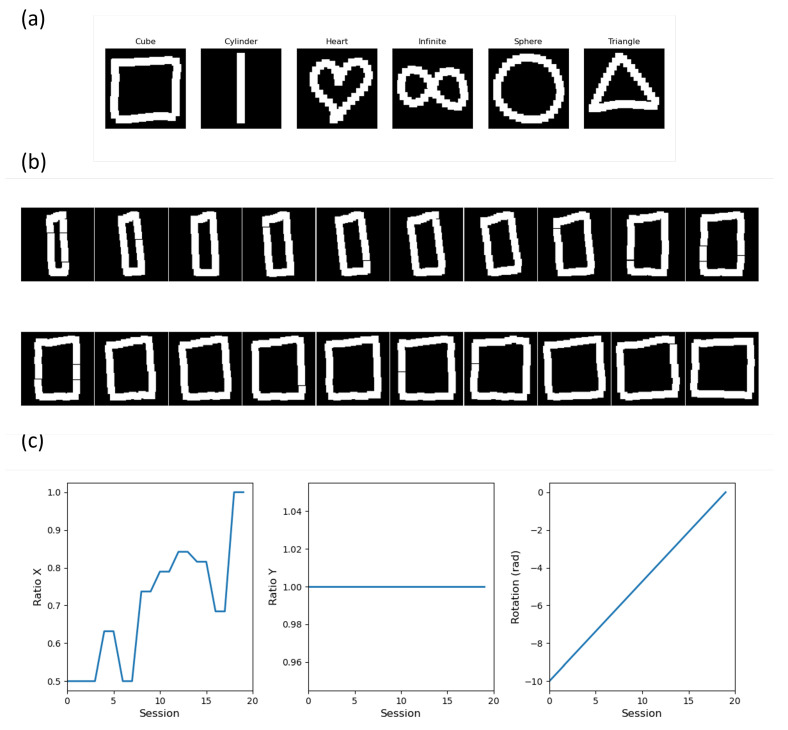
(**a**) Example of each movement recorded with the VR wireless controller. (**b**) Example of artificial data created for the movements “Cube”. A total of 20 movements are created based on the horizontal (Ratio X), vertical stretch (Ratio Y), and rotation factors (**c**).

Data were collected on 10 participants (age: 26.0 (4.1) years, height: 1.70 (0.07) m, mass: 69.9 (8.3) kg) that had no upper extremity pathology that could affect their ability to perform the movements. They gave informed consent after the purpose and content of the study were explained. They were free to withdraw at any time. The data collected consisted of movements drawn with wireless remote control in a VR environment for 6 different movements as presented in Figure 2, called “Cube”, “Cylinder”, “Heart”, “Infinity”, “Sphere”, and “Triangle”. The motion data were then projected onto the frontal plane facing the HMD. Data were collected from 10 participants. Each movement was collected 3 times for each participant for each session and 3 sessions were performed, thus providing a total of 9 repetitions of each movement per participant. Therefore, the total number of movements collected in the database was 540. Data were collected using Unity 2020.3.26f1 with the Oculus Rift S and resampled to 32 points using the “PDollar Point-Cloud Gesture Recognizer” asset library [29]. Data collected on all participants are freely available in the Mendeley repository at the following: https://dx.doi.org/10.17632/kbbprxr4nw.1 (accessed on 7 June 2022).

### 3.2. Database

Each movement is represented by a matrix $X_{l}\in R^{m\times n}$ with m=32 and n=2. For each movement, their corresponding label $y_{l}\in R^{p}$ with *p* = 6, is represented as a binary one-hot vector. Finally, one dataset $D_{t}={\left\{X_{l},y_{l}\right\}}_{l=1}^{N}$ is created with N=54 representing the total number of movements collected. From this data, various artificial data are created in four different steps.

The first step consists, for each label, of randomly selecting an associated matrix $X_{l}$ in D. Three augmentation parameters, namely vertical stretch, horizontal stretch, and rotation, are considered to create variations in the dataset. Vertical and horizontal stretches are achieved by randomly selecting a starting percentage $p_{v}$ and $p_{h}$, respectively, and a starting rotation θ according to the values presented in Table 1. For each augmentation parameter, 20 points are sampled between $p_{h}$ and 1 for the horizontal stretch, between $p_{v}$ and 1 for the vertical stretch, and between θ and 0 for the rotation. The previous sampling is performed on two different, randomly chosen behaviors, which are the linear and the random staircase. A set of motions created according to the augmentation parameters and the corresponding created data are presented in Figure 2 for the “Cube” motion. In addition, each new artificial matrix generated is then augmented three times by adding random noise on the X and Y-coordinates, resulting in a total number of artificial data created equal to 60 for one label.

The second step is to repeat the first step for each label. All the data are then combined into an artificial dataset named $D_{i}^{{}^{\prime }}={\left\{X_{l},y_{l}\right\}}_{l=1}^{N}$ with N=360. The third step is to repeat the second step a total of 20 times and gather all the data into a database with $D_{\mathrm{index}}^{{}^{\prime }}={\left\{D_{i}^{{}^{\prime }}\right\}}_{i=1}^{N}$ with N=20 (index in $D_{\mathrm{index}}^{{}^{\prime }}$ are referred in Table 1).

Finally, the fourth step consists of repeating the third step for each database presented in Table 1, considering different ranges for the augmentation parameters with $p_{v}$ and $p_{h}$ for the vertical and horizontal stretches and θ for the starting rotation.

### 3.3. Training, Validation, and Test Dataset

Each database is separated into three parts, which correspond to training, validation, and testing data. All dataset $D_{i}^{{}^{\prime }}$ in each database (Table 1) were randomly separated into three parts with a ratio of 70%/15%/15% [30] that corresponds to the 12 $D_{i}^{{}^{\prime }}$ dataset used for the training, 3 $D_{i}$ dataset for the validation, and 3 dataset $D_{i}^{\prime }$ for the model testing. The test set is only used to evaluate the final performance of the selected models, while the training and validation sets are used for model selection.

### 3.4. Autoencoder

Autoencoders (AE) are parametric models primarily used for data dimensionality reduction. They are trained in an unsupervised manner, meaning that no label information is provided, and as a deep learning approach, they can provide the nonlinear mapping. They consist of an artificial neural network (ANN) separated into two parts, the encoder and decoder, connected through a latent space (z). The encoder part, Equation (1), is used to reduce the dimensionality of the input data X. The decoder part, Equation (2), is used to reconstruct the original data at its output ($X^{\prime }\in R^{m}$) from z. The structure of the autoencoder is as follows:

(1) $$f_{\left(Encoder\right)}:X\in R^{m}\to z\in R^{d}$$

(2) $$f_{\left(Decoder\right)}:z\in R^{d}\to X^{\prime }\in R^{m}$$

The number of nodes in the output layer $\left(z\in R^{d}\right)$ of the encoder is set to 2 to create a two-dimensional latent space.

**Figure 3:**
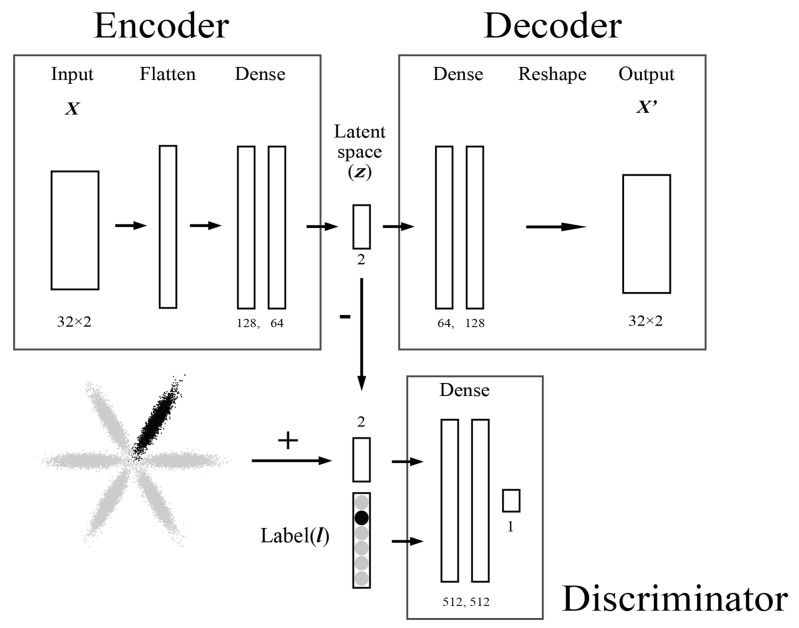
Semi-supervised adversarial autoencoder architecture with ***z*** representing the latent space and ***l*** as the label representing the one-hot vector. The unsupervised adversarial autoencoder will just consist of removing the input ***l***.

The essential information about the input vector z can be represented in a lower-dimensional space using the encoder part. Such an approach allows the distribution of data to be embedded in z, allowing similar input vectors to be close to each other, and thus visually detecting similarities and patterns. To obtain a continuous latent space z, a regularization must be applied to the latent space to shape it in relation to some prior distribution. For this purpose, we consider the adversarial autoencoder model [31]; a diagram of the model is presented in Figure 3.

### 3.5. Adversarial Autoencoder

An adversarial autoencoder (AAE) [31] consists of an autoencoder integrating adversarial training used to constrain the encoding distribution *q*(z|X) to the desired distribution called the prior distribution *p*(z). This provides an *q*(z) aggregated distribution in the latent space that matches *p*(z). This allows a flexible approach for unsupervised and semi-supervised clustering.

The AAE structure is composed of an autoencoder, an adversarial network, Equation (5), composed of the encoder part of the autoencoder, Equation (3), and a discriminator. The discriminator, Equation (4), is connected to the encoder through the latent space z and its output w is a singular node.

The structure of the AAE consists of the same structure as presented in Equations (1) and (2), with the additional constraints as follows:

(3) $$\begin{array}{ccc} & & f_{\left(Autoencoder\right)}:X\in R^{m}\to z\in R^{d}\to X^{\prime }\in R^{m} \end{array}$$

(4) $$\begin{array}{ccc} & & f_{\left(Discriminator\right)}:z\in R^{d}\to w\in R^{1} \end{array}$$

(5) $$\begin{array}{ccc} & & f_{\left(Adversarial\;network\right)}:X\in R^{m}\to z\in R^{d}\to w\in R^{1} \end{array}$$

### 3.6. Adversarial Autoencoder Training

A *K*-Fold cross-validation was performed by considering a *K* =5 folds rotation with 12 and 3 datasets, respectively, for each *K*-fold by selecting $D_{i}^{{}^{\prime }}$ in the training and validation sets randomly (Section 3.3). The validation set is always used to stop training when the considered loss metrics decrease on the test set but increase on the validation set, which would correspond to an over-fitting of the model on the training set. This procedure was used to tune the hyperparameters of the adversarial autoencoder presented in detail in Section 3.8.

At each training epoch, the AAE is trained in two distinct steps called the reconstruction and the adversarial step. In the reconstruction step, the Autoencoder is trained by minimizing the mean square error $L_{AE}$, Equation (6), in order to reconstruct X at the output of the decoder $X^{\prime }$ as follows:

(6) $$L_{AE}=\frac{1}{n}\sum_{i}{\left|X_{i}^{\prime }-X_{i}\right|}^{2}$$

In the adversarial step, the discriminator part of the adversarial network is trained by minimizing the cross-entropy $L_{D}$, Equation (7), to discriminate *p*(z), called the positive sample, from *q*(z), called the negative sample, as follows;

(7) $$L_{D}=-\frac{1}{N}\sum_{n=1}^{N}\left[y_{n}^{\prime }\log\left(y_{n}\right)+\left(1-y_{n}\right)\log\left(1-y_{n}^{\prime }\right)\right]$$

with *q*(z) corresponding to the current distribution in z provided by the encoder. Each sample in z has a corresponding label *y* equal to 0, and each sample in $z^{\prime }$ randomly extracted from the prior distribution *p*(z) has a corresponding label *y* equal to 1. In this step, the parameters of the encoder part of the adversarial network are not updated.

Then, the encoder part of the adversarial network is trained by minimizing $L_{G}$, Equation (8). This step allows the encoder to confuse the discriminator by generating the distribution *q*(z) closer to *p*(z). In this step, the parameters of the discriminator part of the adversarial network are not updated.

(8) $$L_{G}=-\frac{1}{N}\sum_{n=1}^{N}\left[y_{n}^{\prime }\log\left(y_{n}\right)+\left(1-y_{n}\right)\log\left(1-y_{n}^{\prime }\right)\right]$$

Once the training is finished, the encoder becomes a generative model that maps *p*(X) to *p*(z).

In this study, two different approaches are considered. The first one considers an Unsupervised AAE (UAAE) model trained in a fully unsupervised manner with p(z) defined as a set of six two-dimensional Gaussian distributions representing N = 6 movements with the *i*th movement as $z_{i}$∼$N(\mu ,\sigma^{2})$ [24,31]. The second approach considers a Semi-Supervised AAE (SSAAE) approach by integrating the label information at the input of the discriminator Equation (9) during the adversarial phase according to the following modification for the discriminator:

(9) $$\begin{array}{cc} \; & f_{\left(Discriminator\right)}:\left(z,l\right)\in R^{d+N}\to w\in R^{1} \end{array}$$

with l representing the one-shot encoded vector concatenated with z. This approach leads the encoder to confuse the discriminator by integrating each sample into its corresponding $z_{i}$$N(\mu ,\sigma^{2})$. This approach is called semi-supervised since the encoder does not know the labels (only the discriminator does).

The prior distribution used to train the AAE consists of six two-dimensional Gaussian distributions uniformly distributed in a polar coordinate system, and its shape is similar to a polar rose [24].

### 3.7. Latent Space Evaluation

The purpose of using AAE in this study is to generate a latent space where the data points of each movement data can be properly embedded by building an encoder model. It is necessary to perform a proper evaluation of the latent space while training the models. The performance of the models at generating a two-dimensional distribution close to the specified prior distribution is evaluated by considering the average of the Kullback–Leibler (KL) Equation (10) divergence [32] for each two-dimensional Gaussian distribution.

Let p(z) be the prior distribution and q(z) be the output distribution in the latent space for one two-dimensional Gaussian distribution (so p(z) and q(z) are represented as p(z)∼$N(\mu_{p},\Sigma_{p})$ and q(z)∼$N(\mu_{q},\Sigma_{q})$ for each); then the KL divergence is obtained as follows:

(10) $$D_{\mathrm{KL}}\left(p||q\right)=\frac{1}{2}\left(\mathrm{tr}\left(\Sigma_{q}^{-1}\Sigma_{p}\right)+{\left(\mu_{q}-\mu_{p}\right)}^{T}\Sigma_{q}^{-1}\left(\mu_{q}-\mu_{p}\right)-\gamma +\ln\left(\frac{\det\Sigma_{q}}{\det\Sigma_{p}}\right)\right)$$

with γ as the dimension of the distribution. Thus, the mean value of each KL divergence obtained for each cluster, Equation (11), is used as a baseline comparison when training the AAE model as follows:

(11) $$M=\frac{1}{N}\sum_{n=1}^{N}D_{\mathrm{KL},i}$$

with $D_{\mathrm{KL},i}$, the KL divergence of the *i*th movement, and M to be minimized.

### 3.8. Adversarial Autoencoder Hyperparameters

Finding the proper combination of hyperparameters is important because deep learning models can have different degrees of performance depending on their hyperparameters. Various combinations of hyperparameters were tested during a hyperparameter search performed by a genetic algorithm (GA); studies [24,33] indicate the hyperparameters considered.

Table 2 provides the details of the hyperparameters considered, which are the number of layers, the number of nodes per layer, the activation function of the autoencoder, the activation function of the discriminators, the learning rate, and the dropout rate.

Regarding the combination of the number of layers and the number of nodes in each layer, three different approaches are considered. The first one uses the same number of neurons on each layer, the second one uses a decreasing number of nodes (e.g., 16, 8, 4 nodes for 3 layers), and the last one uses an increasing number of nodes (e.g., 4, 8, 16 nodes for 3 layers). The total number of possible combinations is 4499. The following GA parameters were considered: population size of 50, 10 generations, use of tournament selection to choose 10 individuals to be the parents of the next generation, uniform crossover, and 5% chance of random mutation on each gene. The GA is used during cross-validation of the *K*-Fold on the X database for UAAE, and the hyperparameters found are then used for all models and the database (Table 1). The fitness function considered is the KL divergence described earlier.

Three different activation functions that are sigmoid, Equation (12), hyperbolic tangent (tanh), Equation (13), and rectified linear unit (ReLU), Equation (14), are considered.

(12) $$\begin{array}{c} f\left(z\right)=sigmoid\left(z\right)=\frac{1}{\left(1+e^{-z}\right)} \end{array}$$

(13) $$\begin{array}{c} f\left(z\right)=tanh\left(z\right)=\frac{\left(e^{z}-e^{-z}\right)}{\left(e^{z}+e^{-z}\right)} \end{array}$$

(14) $$\begin{array}{c} f\left(z\right)=\left\{\begin{array}{c} 0\;if\;z<0\\ z\;if\;z\geq 0 \end{array}\right. \end{array}$$

The AAE model was trained on a mini-batch size of 64, with the Adam optimizer used to minimize the loss function, Equations (6)–(8). Keras 2.7 [34] was used with Python 3.8 to build the AAE models.

The architecture of the selected models is presented in detail for the SSAAE in Figure 3. One AAE model with the same architecture is built for each database (Table 1).

### 3.9. Latent Space Distance

The database, $D_{t}$, representing the collected data are passed through the trained encoder model to get their representation in the two-dimensional latent space. For each label, a $c_{l}$ centroid called “target centroid” is computed by considering the average coordinates of all points representing a specific label.

For each database, each sample in the testing set (Section 3.3) is then passed through the trained encoder to represent it as a two-dimensional point $z\in R^{d}$ with *d* = 2, providing a new representation of the data. Then, a distance metric, $d_{l}$, Equation (15), for each point z is computed as follows by considering their corresponding centroid $c_{l}$:

(15) $$d_{l}=\sqrt{{\left(z_{l}-c_{n}\right)}^{2}}$$

Then, by repeating this distance calculation for each label, a new dataset $R_{i}={\left\{d_{l},y_{l}\right\}}_{l=1}^{N}$ representing the *i*th artificial subject from the testing set is created. The procedure is repeated for all databases $D_{\mathrm{index}}^{{}^{\prime }}$ (Table 1).

### 3.10. Latent Space Accuracy

For each database, the accuracy of the testing set in the latent space $z\in R^{d}$ is evaluated [24]. The k-nearest neighbors classifier is used to associate each test point with its k-nearest training points with *K* =10. The predicted class ${\hat{y}}_{l}$ is then evaluated by a majority vote considering the most common class among the neighbors and compared to the true label $y_{l}$.

## 4. Multi-Armed Bandit Problem

The RL approach in a VR game for rehabilitation would be beneficial to maintaining a game flow that is neither too difficult nor too easy. RL approaches define a framework between an agent and its environment with respect to states, actions, and rewards [35].

MAB [36] is a specific form of RL that allows exploration and exploitation of an environment without changing its state. By repeatedly updating its internal knowledge with new data (e.g., new records during VR games), the model is constantly kept up to date. MAB does not require large amounts of data, allowing the model to be effective after only a few iterations. The reason for not using a full RL algorithm such as Q-Learning is that the set of possible states and transition probabilities in a Markov decision process cannot be determined at this stage.

In this study, a non-stationary stochastic MAB problem [37,38] is considered to solve sequential decisions during VR games. The MAB problem is considered as a decision-making agent with one state and K=6 arms representing the numbers of movements.

At each iteration t=1,2,...,60, among each arm $K\in R^{n}$ with n=6, the agent chooses an arm k∈K as an action and receives a reward $R_{k}\left(t\right)$ associated with this arm. Then, the agent updates its internal knowledge about the expected reward ${\hat{\mu }}_{k}\left(t\right)$ and chooses a new action accordingly.

${\hat{\mu }}_{k}\left(t\right)$ denotes the expected reward that is estimated sequentially on the basis of the observed reward $R_{k}\left(t\right)$ in order for the agent to keep track of changes in the state. $R_{k}\left(t\right)$ is considered as the distance in the latent space as described in the previous section, which will update the agent’s internal knowledge based on the patient’s motor skills.

The arm selection probability is specifically used here since the goal is not only to select the arm that will provide the highest reward but to select the arms according to the stochastic vector P(t), which represents the relationship with the arm selection and is obtained using the softmax activation function on the vector $\hat{\mu }\left(t\right)$. The updated internal knowledge becomes prior knowledge for the next iteration. The weapons are selected in a probabilistic way (i.e., selection rule) with the objective of adjusting the agent’s knowledge and adapting (i.e., update rule) its decision. The probability is decreased if the reward is small (small distance in the latent space representing a movement close to a target) and increased if it is large (large distance in the latent space representing a movement far from a target). Then, the softmax activation function, Equation (16), is considered as the basis of the selection rule to create P(t) as follows:

(16) $$\begin{array}{c} P_{k}\left(t\right)=\frac{\exp({\hat{\mu }}_{k}\left(t\right)/\tau )}{\sum_{j=1}^{K}\exp({\hat{\mu }}_{k^{{}^{\prime }}}\left(t\right)/\tau )} \end{array}$$

This selection rule formulates the probability of each arm as a function of their distance in latent space. This provides a meaningful way for the agent to not only focus on the maximum reward but also to consider options with lower expectations. The ratio of the exploration/exploitation trade-off varies as a function of the temperature, called τ. As τ increases, selection becomes milder, and probabilities become more uniform (more exploration). Conversely, when τ approaches zero, selection approaches are at a maximum (more exploitation).

Two different MAB exploration methods are considered: Boltzmann (Section 4.1) and Sibling Kalman Filter (Section 4.2). Details on how the models select an action and are updated at each iteration are described in the following sections.

### 4.1. Boltzmann

The Boltzmann model is a model-free method for non-stationary MAB [35]. In a non-stationary MAB problem, the rewards will vary over time. In this case, the method gives more weight to rewards received more recently than to those in the past. After receiving a reward, the agent’s internal knowledge is updated following Equation (17), where the learning rate α is a constant value of 0<α≤1.

(17) $$\begin{array}{c} {\hat{\mu }}_{k}\left(t+1\right)={\hat{\mu }}_{k}\left(t\right)+\alpha \left(R_{k}\left(t\right)-{\hat{\mu }}_{k}\left(t\right)\right) \end{array}$$

#### Upper Confidence Bound (UCB)

The Upper Confidence Bound (UCB) [35,39] consists of adding uncertainties about action-value estimation by adding an uncertainty vector to the expected reward vector, Equation (18). This allows the trade-off ratio to vary according to the uncertainty, increasing the proportion of exploration. Every time an arm, *k*, is explored, the uncertainty decreases with the number of attempts, $N_{k}\left(t\right)$. The degree of exploration can be adjusted by a constant value c>0, providing a new expected reward vector as presented as follows:

(18) $$\begin{array}{c} {\hat{\mu }}_{k}\left(t\right)={\hat{\mu }}_{k}\left(t\right)+c\sqrt{\frac{\log t}{2N_{k}\left(t\right)}} \end{array}$$

Boltzmann exploration is an interesting approach for tracking non-stationary problems, as well as for selecting an arm based on a stochastic vector. A problem remains: the learning rate is not adaptive to the received and expected reward. Indeed, a low learning rate will make the model take several iterations to adapt to potential abrupt changes. Conversely, a high learning rate will make the model more sensitive to noise in the reward. In addition, UCB only increases the degree of exploration at the beginning, and constant updating of the uncertainty of the unselected arms would be useful. To overcome this problem, a state-of-the-art model called the Sibling Kalman Filter is implemented and compared to the Boltzmann model.

### 4.2. Sibling Kalman Filter

The Sibling Kalman Filter model [38,40,41] is an alternative option to the Boltzmann model that varies at each iteration according to the degree of uncertainty on the expected rewards and the variance of the environment in which the agent is operating.

At iteration *t*, the Kalman Filter [42,43] provides posterior distributions associated with the reward $R_{k}\left(t\right)$ for each arm, Equation (19). These distributions indicate the time-varying mean of the expected reward, $\mu_{k}\left(t\right)$, as well as the variance associated with the uncertainty of the expectations, $\epsilon_{k}\left(t\right)$ (i.e., degree of variability in the patient’s actual motor capability and current performance). This uncertainty of the expected reward is sampled from a normal distribution with zero mean and variance $\sigma_{\epsilon }^{2}$, called “observation variance”, as follows:

(19) $$\begin{array}{ccc} R_{k}\left(t\right) & =\mu_{k}\left(t\right)+\epsilon_{k}\left(t\right) & \epsilon_{k}\left(t\right)∼N\left(0,\sigma_{\epsilon }^{2}\right) \end{array}$$

Moreover, since $\mu_{k}$ varies over time, the uncertainty $\xi_{k}\left(t\right)$ associated with the untested arm at each iteration *t* is increased in order to reflect potential variations (i.e., degree of change in a patient’s motor capabilities), Equation (20). This uncertainty is also sampled from a normal distribution with zero mean and variance, $\sigma_{\xi }^{2}$, called “innovative variance” as follows:

(20) $$\begin{array}{ccc} \mu_{k}\left(t\right) & =\mu_{k}\left(t\right)+\xi_{k}\left(t\right) & \xi_{k}\left(t\right)∼N\left(0,\sigma_{\xi }^{2}\right) \end{array}$$

The acquisition of two unique variances is adapted by parameters.

Bayesian estimation of the mean of the expected reward ${\hat{\mu }}_{j}\left(t\right)$ and the associated variance ${\hat{\sigma }}_{j}^{2}\left(t\right)$ is performed by the Kalman filter. These prior distributions are considered to be normally distributed, and the likelihood is considered as being sampled from a normal distribution. Thus, the posterior distribution can be represented by the same distribution as the prior.

According to the reward, $R_{k}\left(t\right)$, obtained from the selected arm, the agent’s internal knowledge, $\hat{\mu }\left(t\right)$, Equation (21), is updated with the following updated rule:

(21) $$\begin{array}{c} {\hat{\mu }}_{k}\left(t+1\right)=\left\{\begin{array}{cc} {\hat{\mu }}_{j}\left(t\right)+G_{j}\left(R_{j}\left(t\right)-{\hat{\mu }}_{j}\left(t\right)\right) & if\;Arm\;k=j\\ {\hat{\mu }}_{k}\left(t\right) & otherwise \end{array}\right. \end{array}$$

With $G_{k}\left(t\right)$ being the Kalman gain, Equation (22), used as an adaptive learning rate and computed as follows:

(22) $$\begin{array}{c} G_{k}\left(t+1\right)=\left\{\begin{array}{cc} \frac{{\hat{\sigma }}_{j}^{2}\left(t\right)+\sigma_{\xi }^{2}}{{\hat{\sigma }}_{j}^{2}\left(t\right)+\sigma_{\xi }^{2}+\sigma_{\epsilon }^{2}} & if\;Arm\;k=j\\ 0 & otherwise \end{array}\right. \end{array}$$

Unlike the UCB seen in the previous section with the Boltzmann model, the Sibling Kalman filter addresses the uncertainty problem not only at the beginning but continuously. Thus, the uncertainty of the distribution is updated at each iteration following Equation (23), which reduces the uncertainty with respect to the arm for which the reward is observed and increases it for the untested arms.

(23) $$\begin{array}{c} {\hat{\sigma }}_{k}^{2}\left(t+1\right)=\left\{\begin{array}{cc} \left(1-G_{j}\left(t\right)\right)\left({\hat{\sigma }}_{j}^{2}\left(t\right)+\sigma_{\xi }^{2}\right) & if\;Arm\;k=j\\ {\hat{\sigma }}_{k}^{2}\left(t\right)+\sigma_{\xi }^{2} & otherwise \end{array}\right. \end{array}$$

The first variant of the Sibling Kalman filter considered the use of the softmax activation function, Equation (16), based on the expected reward vectors, ${\hat{\mu }}_{k}\left(t\right)$, Equation (21). Thus, this model only takes into account the adaptive learning rate.

Two other variants of the Sibling Kalman filter that use the innovative upper confidence limit and Thomson sampling are described in the next two sections. Thus, these models consider the adaptive learning rate as well as the uncertainty of the expected rewards.

#### 4.2.1. Innovative Upper Confidence Bound (IUCB)

An Innovative Upper Confidence Bound (IUCB) [40] is here adapted in order to consider the uncertainty of the prior distribution of the estimated reward. Therefore, Equation (24) is used as an alternative to the UCB vector described previously in Equation (18) by considering the uncertainty of the prior distribution as well as the “observation noise”:

(24) $$\begin{array}{c} {\hat{\mu }}_{k}\left(t\right)={\hat{\mu }}_{k}\left(t\right)+c\sqrt{{\hat{\sigma }}_{k}^{2}\left(t-1\right)+\sigma_{\epsilon }^{2}} \end{array}$$

#### 4.2.2. Thomson Sampling (TS)

Thompson Sampling (TS) [38,40,44] is considered a stochastic strategy that samples the estimated reward based on both the updated estimated mean ${\hat{\mu }}_{k}\left(t\right)$ and uncertainty (${\hat{\sigma }}_{k}^{2}\left(t\right)$). Thus, the probability that each arm, *k*, has the highest mean is computed, Equation (25). For each arm, the predicted reward from the prior distribution is sampled, and this sampled value, $\theta_{k}\left(t\right)$, is used as a reference, which is normalized by the softmax activation function to get the stochastic vector P(t) as follows:

(25) $$\begin{array}{ccc} P_{k}\left(t\right) & =\frac{\exp(\theta_{k}\left(t\right)/\tau )}{\sum_{k^{{}^{\prime }}=1}^{K}\exp(\theta_{k^{{}^{\prime }}}\left(t\right)/\tau )} & \theta_{k}\left(t\right)∼N\left({\hat{\mu }}_{k}\left(t\right),{\hat{\sigma }}_{k}^{2}\left(t\right)\right) \end{array}$$

The arm is then selected according to the above-mentioned stochastic vector.

### 4.3. Agent Models

Based on the methods described so far, five models are presented in Table 3 with details on the update and selection rules used and their corresponding equations. The names of these methods are related to the respective update and selection rules: Boltzmann, Boltzmann UCB, Sibling Kalman Filter, Sibling Kalman Filter IUCB, and Sibling Kalman Filter TS. A summary of the required parameters for each method is also presented.

The Boltzmann and Sibling Kalman Filter algorithms are presented in detail in the Appendix A for each method.

### 4.4. Model Evaluation

Usually, the evaluation of MAB models is based on regret, which is defined as the difference between the reward obtained and the highest expected reward. In the present case, the selection of consecutive arms with a high expected value is not required, and the objective is to properly evaluate the probability distribution of the expected reward. Therefore, by adding a metric to these evaluations, this paper attempts to provide an appropriate evaluation method for the results obtained when using the softmax activation function. The metric assesses whether each arm can accurately follow the trajectory obtained from the latent space according to the evaluation criterion based on the softmax activation function.

The following metric is considered by comparing P(t) and $\hat{\mu }\left(t\right)$ with the true probability distribution of the arms and the true rewards denoted by $P{\left(t\right)}^{*}$ and $\hat{\mu }{\left(t\right)}^{*}$, respectively, which corresponds to the absolute difference between the weighted probability accumulated at iteration point *T* as follows:

(26) $$\begin{array}{c} ae\left(T\right)=\sum_{t=0}^{T}\sum_{k=1}^{K}\left|P_{k}{\left(t\right)}^{*}{\hat{\mu }}_{k}{\left(t\right)}^{*}-P_{k}\left(t\right){\hat{\mu }}_{k}\left(t\right)\right| \end{array}$$

with the temperature value in the softmax activation to get the true probability, $P_{k}{\left(t\right)}^{*}$ is set to an empirical value of 2 to favor exploration (further study is needed on this value).

The hyperparameters presented in Table 4 are the initial Q-values $Q_{0}$, the learning rate α, the temperature τ, the confidence level *c*, the innovative variance $\sigma_{\xi }^{2}$, and the observation variance $\sigma_{\epsilon }^{2}$, which are tested using a grid search with the X database. The combination of hyperparameters that minimize Equation (26) were selected and applied to all remaining databases. In addition, Table 4 shows the selected parameters in bold for each model.

## 5. Results

### 5.1. Latent Space

**Figure 4:**
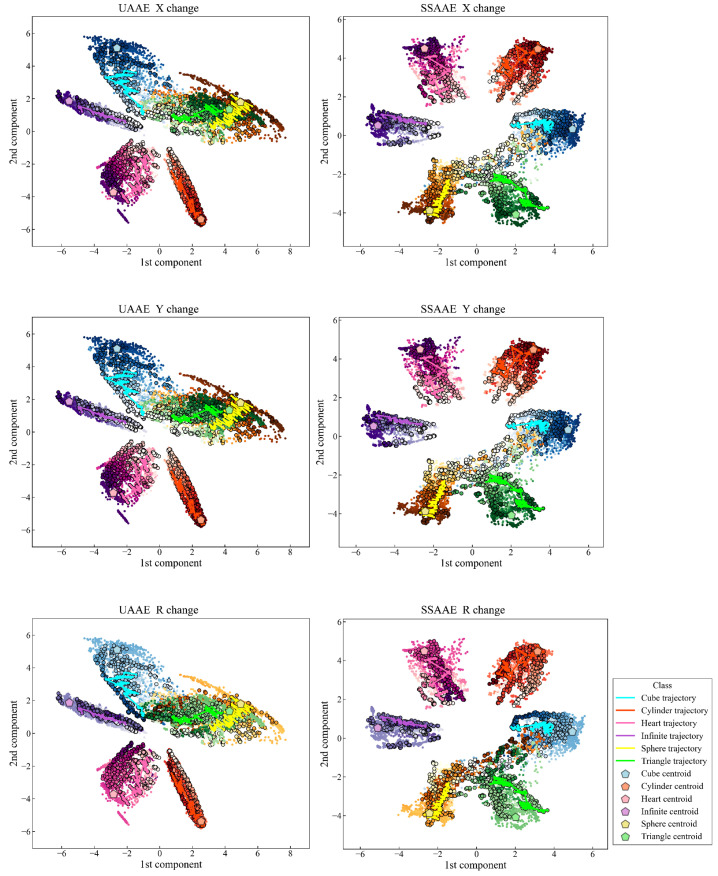
UAAE latent space (**left**) and SAAE latent space (**right**) representation with their corresponding gradient information regarding the “augmented parameters” values for horizontal stretch (**top**), vertical stretch (**middle**), and rotation (**bottom**).

The latent space visualizations obtained with UAAE and SSAAE with the XYR database are presented in Figure 4, with a trajectory for each movement representing the entirety of an artificial data set. In addition, each label is represented by a unique set of colors with a color gradient depending on the selected behavior (horizontal stretch, vertical stretch, or rotation). The darker the color, the closer the artificial data is to the original data. The latent space created is continuous, and the trajectories clearly go to the target represented by the original data of the D dataset. The latent space accuracy was evaluated on the test set (Section 3.10) for each AAE model and averaged across all databases as follows 87.9% (6.9%) and 97.9% (2.2%) for UAAE and SSAAE, respectively. The detailed results are as follows for UAAE: 100.0%, 85.7%, 95.8%, 79.4%, 88.0%, 82.8%, and 83.6% for databases X, Y, R, XY, XR, YR, and XYR, respectively. Regarding SSAAE, the results are as follows: 100.0%, 99.9%, 99.1%, 98.7%, 98.7%, 94.6%, and 94.4%. Figure 4 shows the different latent spaces obtained by UAAE and SSAAE. SSAAE produced a better latent space compared to the unsupervised one. This is due to the fact that SSAAE uses the label information during the adversarial phase, which allows the corresponding movement to be placed in its respective cluster more efficiently. Indeed, the latent space of UAAE has two clusters (Sphere and Triangle) that are completely entangled. AAE provides better results if it is guided during the adversarial phase by considering a semi-supervised approach to find an optimal solution for mapping the data distribution to the prior distribution [24].

### 5.2. Multi-Armed Bandit Problem

**Figure 5:**
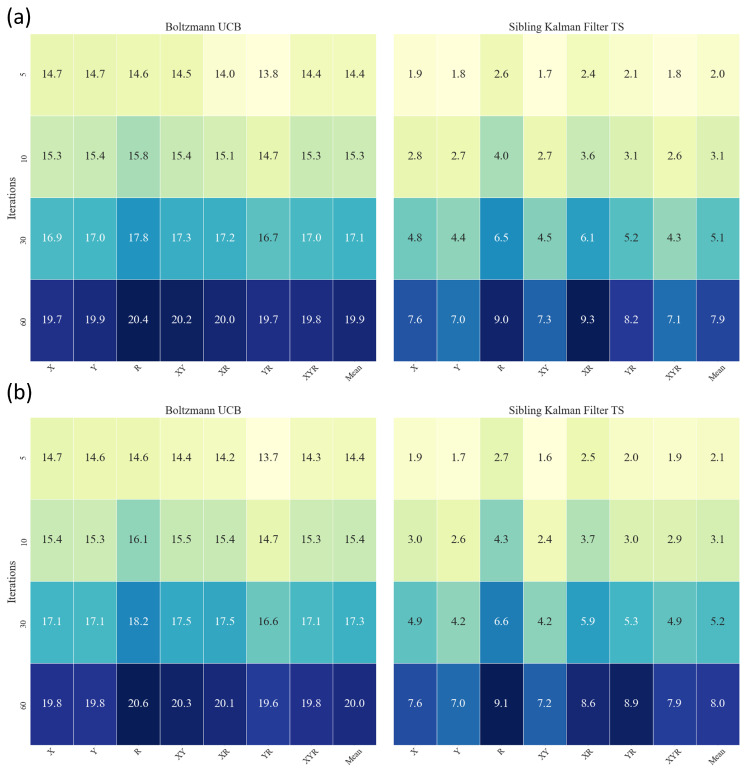
MAB Heatmap showing the results for UAAE (**a**) and for SSAAE (**b**) at iterations 5, 10, 30, and 60 for Boltzmann UCB (left) and Sibling Kalman Filter with Thomson Sampling (right) for each database (X, Y, R, XY, XR, YR, XYR).

**Figure 6:**
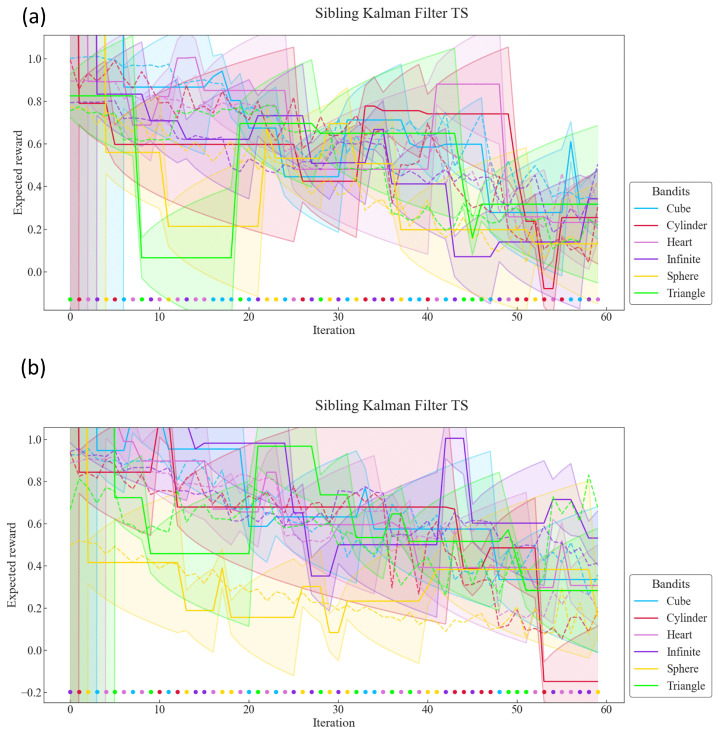
MAB results for UAAE (**a**) and for SSAAE (**b**) for the Sibling Kalman Filter with Thomson Sampling for all iterations on one artificial test dataset from the database XYR. The colored area represents the uncertainty ${\hat{\sigma }}_{k}{\left(t\right)}^{2}$ about the expected reward ${\hat{\mu }}_{k}$.

Results for the metrics of Equation (26) are presented in Figure 5 at iterations 5, 10, 30, and 60 for all databases. The Boltzmann UCB and Sibling Kalman Filter TS variants are specifically presented as they had the lowest cumulative error in their respective categories. The Sibling Kalman Filter exploration outperformed the Boltzmann exploration at all iterations. For example, the results showed, at iteration 60, a weighted average cumulative probability error for all databases of 7.9 versus 19.9 using the UAAE latent space representation and 8.0 versus 20.0 using the SSAAE latent space representation. In addition, a detailed graph representing the MAB results for the Sibling Kalman filter with Thomson sampling for all iterations on an artificial data set in $D_{xyr}$ is presented in Figure 6. The results presented in Figure 4 and Figure 6 are from the same artificial data set.

## 6. Discussion

This paper aims to investigate data dimensionality reduction models using models called adversarial autoencoders to provide an effective visualization tool for high dimensional data. Based on hand movements performed with wireless controllers in an immersive VR environment, AAE models were tested in unsupervised and semi-supervised contexts to represent each movement as a single two-dimensional point. This visualization can be effectively used to detect outliers and trajectories (patterns) for the rapid analysis and interpretation of novel motions. In addition, the latent space representations of the data were used to represent the current representation of the motion relative to a target to compute a distance measure used for a specific class of reinforcement learning model, called Multi-armed Bandit.

Considering an increasing complexity, seven different databases, consisting of 20 artificial datasets each, were created from the collected data based on various factors, such as horizontal stretch, vertical stretch, and rotation. In addition, random noise was used as a data augmentation approach to increase the generalization performance of the deep learning model [45].

To ensure that the latent space representation provides a correct representation based on the prior distribution, the KL divergence was used as a fitness function in a genetic algorithm to find a good set of hyperparameters that can help create such a match. As shown in Figure 4, the latent space provides a cluster for each label with a homogeneous size by following several Gaussian distributions in the latent space. This allows a clear visualization and provides a way to numerically facilitate the comparison between different movements by reducing an imbalance in the calculated distance in each cluster. The accuracy of the test set was evaluated, and the results showed an average accuracy for all databases of 87.9% (6.9%) and 97.9% (2.2%) for UAAE and SSAAE, respectively. As shown in Figure 4, two clusters are entangled for UAAE, which reduces the accuracy. This may be due to the fact that the data in these areas are highly distorted, making it difficult for an unsupervised approach to properly separate the two movements. Using a semi-supervised approach allows AAE to focus on the essential parts of the signal to correctly separate the signals in the latent space. Nevertheless, AAE can still perform well and be an interesting approach if no label information is available. It should be noted that incorporating label information in the adversarial phase does not significantly increase the complexity of the models, and if labeled data are available, a semi-supervised approach should be preferred. Furthermore, as presented in Figure 4, trajectories representing a full set of artificial $D_{i}$ data can be observed clearly. Obtaining such results with data corresponding to a patient’s progression through various VR game sessions during rehabilitation would greatly assist clinicians in interpreting a large amount of data. This will speed up the analysis of improvement and the current status of the patient. It should be noted that even if the amount of data (360 artificial data) considered in each database is small, the AAE models are still able to efficiently generalize the movement, as shown by the accuracy of the test sets. As stated in [46], there is no specific threshold regarding the amount of data required to use deep learning, as it is related to the task being solved and the size of the neural network used for it.

The test set points in the latent space are then represented by a distance measure from a target. The target corresponds to the centroids computed from the data collected from the healthy participants. These distances are then used as a reward in the MAB algorithm so that the agent learns the current distance in latent space and thus provides a probabilistic decision base for the DDA to adapt the game to the patient’s motor capabilities.

The objective is to adjust the agent’s knowledge and adapt its decision by reducing the probability of choosing an action if the reward is low (good movement represented by a small distance) and by increasing it if the reward is high (bad movement represented by a large distance). However, other movements must be tested continuously to maintain the patient’s motivation and to know their evolution (new distances in the latent space). In other words, the ratio of these trade-offs allows us to generate a probability sequence for selecting the next movement. The softmax activation function is used to enable probabilistic selection, and the ratio of trade-offs is defined by the temperature parameter. Thus, the agent can adapt the game by selecting moves that are less likely, or more likely, to make the patient win based on their motor control capability.

Figure 5 and Figure 6 show that the Sibling Kalman filter with Thomson sampling outperforms the Boltzmann exploration with UCB. Indeed, the results on the cumulative error at the end of the episode (iteration 60) are two times smaller for the Sibling Kalman filter with Thomson sampling compared to the Boltzmann model. Moreover, Sibling Kalman shows a faster adaptation at the beginning with, for example, an average error of 2.1 at iteration five compared to 14.4 with Boltzmann exploration.

Kalman filter models have two important advantages over Boltzmann exploration, which is important in a rapidly changing environment to provide an efficient probability-based decision model. The first is an adaptive learning rate based on the current difference between the reward and the expected reward and the estimated variance of the data. The second is that an additional degree of exploration is considered at each iteration where an action is not tested. MAB agents based on these models appear to be particularly well suited to a rapidly changing non-stationary environment, which is of interest in DDA rehabilitation games. In addition, the MAB is initialized with Q-optimistic values, but these values can be chosen based on past data based on knowledge of past patients. This can allow the MAB agent to update its internal knowledge of the condition more accurately for new participants.

## 7. Limitations

It is important to note that the main limitation of this study is the data. Indeed, further studies need to investigate whether the use of artificial data can ease the problem of collecting enough data to create such complex deep learning models. In addition, real patient data should also be tested and verify whether similar trajectories emerge during certain clinician-guided rehabilitation processes. In any case, end effector motion data, combined with effective data dimensionality reduction models, appear to have the potential to enable the development of effective visualization tools.

Another limitation regarding the internal knowledge of the MAB agent is that it can be used to make decisions based on adjusted probabilities by changing the temperature value. A large temperature value makes the difference between the milder probability, while a value closer to zero gives more weight to the higher expected reward. Further experimentation is needed to find a good temperature value that will provide the best experience, neither too easy nor too difficult, for patients in real-world rehabilitation settings. Finally, movements that are too difficult or too easy should also be detected and replaced if necessary, which was not considered in this study.

## 8. Conclusions

In conclusion, the use of data dimensionality reduction to create a two-dimensional latent visualization space combined with a reinforcement learning model based on the Multi-armed Bandit algorithm provides an interesting approach to visualize and track current motor control capabilities regarding a target. For example, it is possible to quickly detect abnormal movements by visual inspection. In addition, this simplified data representation can also be used as a way to show the patient his or her progress and to help in the motivation process.

Indeed, movements that would not be correctly executed due to poor motor control capabilities can be easily and visually detected in latent space and used in combination with RL algorithms with the goal of implementing an agent that learns from the environment. MAB agents update information about each arm as they are rewarded and make the next decision. Thus, the agent continuously monitors the patient’s ability to perform movements based on the latent space representation and adapts the probability of selecting the next action accordingly.

The results showed that AAE models can represent highly dimensional data in a two-dimensional latent space and that MAB agents can efficiently and quickly learn the evolution of distance in the latent space. This could be applied in the context of DDA to reflect the patient’s abilities in VR games by adapting the game parameters (e.g., changing the properties of an attack or healing) accordingly. This would also be of interest in the context of DDA to reflect the patient’s abilities in VR games. The use of VR games in a home rehabilitation training setting with such approaches could provide interesting complementary tools for the clinician to remotely assess patient progress.

## Figures and Tables

**Table 1:** Parameters considered for the creation of the various artificial databases. **X** represents the range of factors for the vertical stretch, **Y** represents the range of factors for the horizontal stretch, and **R** represents the range of factors for the rotation. Each database contained 20 artificial datasets. Each dataset contained 60 matrices for each label, thus 360 matrices in total.

Database	X	Y	R
$D_{x}^{{}^{\prime }}$	10≥ $p_{h}$ ≥50	90≥ $p_{v}$ ≥100	−5≥ θ ≥5
$D_{y}^{{}^{\prime }}$	90≥ $p_{h}$ ≥100	10≥ $p_{v}$ ≥50	−5≥ θ ≥5
$D_{r}^{{}^{\prime }}$	90≥ $p_{h}$ ≥100	90≥ $p_{v}$ ≥100	−45≥ θ ≥45
$D_{\mathrm{xy}}^{{}^{\prime }}$	10≥ $p_{h}$ ≥50	10≥ $p_{v}$ ≥50	−5≥ θ ≥5
$D_{\mathrm{xr}}^{{}^{\prime }}$	10≥ $p_{h}$ ≥50	90≥ $p_{v}$ ≥100	−45≥ θ ≥45
$D_{\mathrm{yr}}^{{}^{\prime }}$	90≥ $p_{h}$ ≥100	10≥ $p_{v}$ ≥50	−45≥ θ ≥45
$D_{\mathrm{xyr}}^{{}^{\prime }}$	10≥ $p_{h}$ ≥50	10≥ $p_{v}$ ≥50	−45≥ θ ≥45

**Table 2:** Tested hyperparameters.

Hidden Layers	Dense Size	Autoencoder Activation	Discriminator Activation	Learning Rate	Dropout Rate
	4, 8, 16, 32,	Sigmoid, ReLU	Sigmoid	0.01, 0.005, 0.001,	0, 0.1, 0.2,
2, 3	64, 128, 256, 512	Tanh	ReLU	0.0005, 0.0001	0.3, 0.4

**Table 3:** The models for comparative validation.

Model	Update Rule	Selection Rule	Parameters
Boltzmann	Learning rate	softmax choice: Equation (16)	optimistic Q: $Q_{0}$ learning rate: α temperature: τ
Boltzmann UCB	Learning rate	softmax choice with UCB vector: Equations (16) and (18)	optimistic Q: $Q_{0}$ learning rate: α confidence level: *c* temperature: τ
Sibling Kalman Filter	Kalman gain: Equation (22)	softmax choice: Equation (16)	optimistic Q: $Q_{0}$ innovative variance: $\sigma_{\xi }^{2}$ observation variance: $\sigma_{\epsilon }^{2}$ temperature: τ
Sibling Kalman Filter IUCB	Kalman gain: Equation (22)	softmax choice with IUCB vector: Equations (16) and (24)	optimistic Q: $Q_{0}$ innovative variance: $\sigma_{\xi }^{2}$ observation variance: $\sigma_{\epsilon }^{2}$ temperature: τ confidence level: *c*
Sibling Kalman Filter TS	Kalman gain: Equation (22)	softmax choice on TS on Normal distribution: Equation (25)	optimistic Q: $Q_{0}$ innovative variance: $\sigma_{\xi }^{2}$ observation variance: $\sigma_{\epsilon }^{2}$ temperature: τ

**Table 4:** List of hyperparameters. The selected hyperparameters are in bold.

Model	Parameters List
Boltzmann	$Q_{0}$ = [5]
	α = [0.05,0.1,0.2,0.5,1.0]
	τ = [1,2,3]
Boltzmann UCB	$Q_{0}$ = [5]
	α = [0.05,0.1,0.2,0.5,1.0]
	*c* = [1,2,3]
	τ = [1,2,3]
Sibling Kalman Filter	$Q_{0}$ = [5]
	$\sigma_{\xi }^{2}$ = [0.01,0.05,0.1,0.2,0.5,1,2]
	$\sigma_{\epsilon }^{2}$ = [0.01,0.05,0.1,0.5,1]
	τ = [1,2,3]
Sibling Kalman Filter IUCB	$Q_{0}$ = [5]
	$\sigma_{\xi }^{2}$ = [0.01,0.05,0.1,0.2,0.5,1,2]
	$\sigma_{\epsilon }^{2}$ = [0.01,0.05,0.1,0.5,1]
	τ = [1,2,3,4]
	*c* = [1,2,3,4]
Sibling Kalman Filter TS	$Q_{0}$ = [5]
	$\sigma_{\xi }^{2}$ = [0.01,0.05,0.1,0.2,0.5,1,2]
	$\sigma_{\epsilon }^{2}$ = [0.01,0.05,0.1,0.5,1]
	τ = [1,2,3,4]

## Data Availability

Data collected on all participants are freely available in the Mendeley repository at the following: https://dx.doi.org/10.17632/kbbprxr4nw.1 (accessed on 7 June 2022).

## Abbreviations

    The following abbreviations are used in this manuscript:

VR	Virtual Reality
HMD	Head-Mounted Display
RL	Reinforcement Learning
DDA	Dynamic Difficulty Adjustment
MAB	Multi-Armed Bandits
AAE	Adversarial Autoencoder
UAAE	Unsupervised Adversarial Autoencoder
SSAAE	Semi-Supervised Adversarial Autoencoder
UCB	Upper Confidence Bound
IUCB	Innovative Upper Confidence Bound
TS	Thomson Sampling

## Appendix A

**Algorithm A1:** MAB Algorithm—Boltzmann
**Parameter:**$T=60,K=6,Q_{0},\alpha ,\tau ,c$ **Initialization:** Set $\hat{\mu }\left(0\right):=Q_{0}$, N(0):=0 1: **for** t=0,1,⋯,T **:** 2: **if** Boltzmann: 3: k← Softmax choice ($\hat{\mu }\left(t\right),\tau$) with Equation (16) 4: **else if** Boltzmann UCB: 5: k← Softmax choice with UCB vector ($\hat{\mu }\left(t\right),\tau$) with Equations (16) and (18) 6: $R_{k}\left(t\right)\leftarrow$ Bandits (*k*) 7: ${\hat{\mu }}_{k}\left(t+1\right)\leftarrow$ Update rule - Learning rate (${\hat{\mu }}_{k}\left(t\right),R_{k}\left(t\right),\alpha$) with Equation (17)

**Algorithm A2:** MAB Algorithm—Sibling Kalman Filter
**Parameter:**$T=60,K=6,Q_{0},\tau ,c,\sigma_{\xi }^{2},\sigma_{\epsilon }^{2}$ **Initialization:** Set $\hat{\mu }\left(0\right):=Q_{0}$, ${\hat{\sigma }}^{2}\left(0\right),N\left(0\right):=0$ 1: **for** t=0,1,⋯,T **:** 2: **if** Sibling Kalman Filter **:** 3: k← Softmax choice ($\hat{\mu }\left(t\right),\tau$) with Equation (16) 4: **else if** Sibling Kalman Filter IUCB: 5: k← Softmax choice with IUCB vector ($\hat{\mu }\left(t\right),\tau ,c$) with Equations (16) and (24) 6: **else if** Sibling Kalman Filter TS: 7: k← Softmax choice on TS ($\hat{\mu }\left(t\right),\tau$) with Equation (25) 8: $R_{k}\left(t\right)\leftarrow$ Bandits(*j*) 9: $G_{k}\left(t\right),{\hat{\mu }}_{j}\left(t+1\right),{\hat{\sigma }}^{2}\left(t+1\right)\leftarrow$ Update rule - Kalman gain (${\hat{\mu }}_{k}\left(t\right),{\hat{\sigma }}^{2}\left(t\right),R_{k}\left(t\right),\sigma_{\xi }^{2},\sigma_{\epsilon }^{2},k$) with Equations (21)–(23)
